# Identification of biomarkers, pathways and potential therapeutic agents for salt-sensitive hypertension using RNA-seq

**DOI:** 10.3389/fcvm.2022.963744

**Published:** 2022-08-10

**Authors:** Xiaoying Chao, Zhiyuan Jiang, Guoqiang Zhong, Rongjie Huang

**Affiliations:** ^1^Department of Cardiology, The First Affiliated Hospital of Guangxi Medical University, Nanning, China; ^2^Division of Hypertension, The First Affiliated Hospital of Guangxi Medical University, Nanning, China

**Keywords:** RNA-sequencing, differentially expressed genes, biomarker, salt-sensitive hypertension, salt-resistant hypertension

## Abstract

**Background:**

Salt-sensitive hypertension (SSH) is a common type of essential hypertension in China. In recent years, although an increasing number of researches have focused on SSH, few studies have been researched on patients with SSH. The objective of this study was to explore the genes and pathways linked with SSH using RNA-sequencing (RNA-seq).

**Materials and methods:**

We used RNA-seq to analyze the transcriptome of peripheral blood mononuclear cells (PBMCs) of five SSH patients and five SRH patients. Next, we analyzed the differentially expressed genes (DEGs) using Gene Ontology (GO), Kyoto Encyclopedia of Genes and Genomes (KEGG) pathway, and Gene Set Enrichment (GSEA) enrichment analysis. Then, Cytoscape was used to construct the protein-protein interaction (PPI) network and the hub genes. Finally, CMAP analysis found that several small molecular compounds could reverse the altered DEGs.

**Results:**

A total of 431 DEGs were found in the PBMC samples, including 294 up-regulated and 137 down-regulated genes. Functional enrichment analysis found significant enrichment in immune-related associations such as inflammation, chemokine, and cytokine-cytokine receptor interaction. The hub genes of the two modules were IL-6, IL-1A, CCL2, CCL3L3, and BUB1. In addition, we identified two small molecular compounds (iopromide and iloprost) that potentially interacted with DEGs.

**Conclusion:**

This study suggests some potential biomarkers for the diagnosis of SSH. It provides new insights into SSH diagnosis and possible future clinical treatment.

## Introduction

Hypertension is a major cause of premature death worldwide. It is a risk factor for cardio-cerebral vascular disease and kidney disease (1–3). The study estimated that more than 1.2 billion adults worldwide had hypertension in 2019, twice the number of hypertension patients in 1990 (4). The total number of patients with hypertension in the population of 18 years and older in China is 244 million, and the prevalence of hypertension is about 27.9%. Based on this calculation, about one in every four adults is a hypertensive patient (5). High salt intake is a key risk factor for the onset of hypertension, and increasing salt intake promotes the expansion of extracellular fluid volume and increases cardiac output (6). However, different individuals have different effects of salt intake on blood pressure. In the 1970s, Luft and Kawasaki successively found that some hypertensive patients had abnormal blood pressure in acute and chronic salt load tests, and proposed the concept of salt-sensitive hypertension (SSH) (7, 8). Those whose blood pressure rises significantly after salt loading are called salt-sensitive people and those whose blood pressure does not rise or drop significantly are called salt-resistant people (9, 10). The increase in salt intake of salt-sensitive rats is higher than that of salt-resistant rats. The increased salt intake of salt-sensitive rats can also cause organ damage, such as cardiac hypertrophy, vascular hypertrophy, and nephrosclerosis (11). The recent finding showed that the gut microbiota might act as a potential target to counteract SSH (12). To date there has been little agreement on what causes salt sensitivity, so unlocking the molecular secrets of salt sensitivity is a crucial health goal. He et al. reported that salt sensitivity is a cause, not a consequence of hypertension (6). However, it is unclear differences and potential therapeutic targets in SSH and salt-resistant hypertension (SRH).

In recent years, an increasing of researches have demonstrated that transcriptomic changes in peripheral blood can serve as biomarkers of diseases (13, 14). Peripheral blood mononuclear cells (PBMCs) include monocytes and lymphocytes. There is a high correlation between the gene expression profiles of tissues and the corresponding PBMCs (15). In addition, plasma is more readily available than tissue, and it can be used to detect disease status and treatment effects.

RNA-seq has been reported on the potential molecular etiology or therapeutic targets in a variety of cardiovascular diseases, including atherosclerotic, atrial fibrillation, and heart failure (16–18). Nevertheless, there are no reports on the application of RNA-seq in human SSH and SRH study.

In our study, we used RNA-seq to identify those differentially expressed genes in PBMCs from SSH and SRH patients and then analyzed using a variety of bioinformatics methods. Subsequently, several hub genes and potential therapeutic compounds were found. The study may help to discover the underlying pathogenesis of SSH, better understand the etiology of SSH and even develop more effective and targeted treatment of SSH in the future.

## Materials and methods

### Patient studies

For this study, a total of 72 patients with a history of hypertension were selected and peripheral blood samples were collected from February 2021 to August 2021 at the Department of Cardiology, the First Affiliated Hospital of Guangxi Medical University. The informed consent forms used in this study were approved by the Ethics Committee of the first affiliated Hospital of Guangxi Medical University. All experiments were performed by the principles and regulations established by the ethics committee.

All the participants were registered according to the following inclusion criteria: (a) diagnosis of essential hypertension, and the diagnostic criteria of definite SSH as previously reported by Citterio et al. (19). (b) Age between 30 and 70 years, (c) while the exclusion criteria were: individuals with severe diabetes, heart failure, stroke, peripheral artery disease, congenital heart disease, acute myocardial infarction, liver and kidney disease, or cancer patients. All 72 patients were categorized into two groups including SSH group with 29 patients and SRH group have 43. A modified Sullivan acute NS-loading test was used to distinguish SRH and SSH patients, and mean arterial pressure (MAP) was also measured with the systolic blood pressure (SBP) and diastolic blood pressure (DBP) for each time point. Blood pressure was measured at two-time points: baseline BP (before and after sodium-loading). After sodium-loading, two times for each MAP value (MAP1 and MAP2) were calculated from the corresponding 2 BP values. MAP was considered to be the DBP plus one-third of pulse pressure (PP). Patients with MAP2 − MAP1 ≥ 5 mm Hg were diagnosed with SSH and others with SRH. This diagnostic method has widely been used to differentiate the SSH from SRH and was also validated to have the same accuracy as the long-period sodium-loading test. Furthermore, five patients from each group were randomly selected for subsequent RNA-seq analysis.

### Total RNA extraction and library construction

Under aseptic conditions, the collected whole blood was mixed with an equal volume of PBS, and Ficoll-Hypaque density gradient centrifugation was used to isolate the peripheral blood mononuclear cells (PBMC). Then, TRIzol reagent (Invitrogen) was added to the PBMC at a ratio of about 1 × 10^7^ cells/1.5 ml to degrade the proteins and the RNA was extracted from the cells. In addition, the Agilent 2100 Bioanalyzer (Agilent DNA 1000 Reagents) was also used to estimate the total RNA concentration, RNA integrity number (RIN), 28S/18S, and fragment size to evaluate the yield, purity, and integrity of RNA.

### RNA sequencing

MGISEQ-2000 sequencing technology was being used for RNA-Seq sequencing. The qualified library was denatured into the single-stranded library by adding NaOH and then the solution was diluted to a certain concentration according to the expected amount of data on the machine. Afterward, the denatured and diluted library was loaded to Flow Cell for hybridization with the adapter, and then the cluster generation platform cBot was used to complete the bridge PCR amplification. Finally, the prepared Flow Cell was sequenced using Illumina sequencing system SBS reagents. The sequencing data was aligned to the reference gene sequence (reference species: Homo sapiens; data source: NCBI; reference genome version: GCF_000001405.39_GRCh38.p13) for clean read using Bowtie2 (version: v2.2.5) (20) and RSEM (Version: v1.2.8) (21) was used to calculate the gene expression levels of each sample.

### Sample correlation analysis and differential gene identification

DESeq2 package (version 1.36.0) in R software (version 3.6.0) for estimate variance-mean dependence in count data from high-throughput sequencing assays and test for differential expression based on a model using the negative binomial distribution (22). The FDR < 0.05 and |log2 (fold change)| > 1 values were used as a criterion to find the differentially expressed genes (DEGs). In addition, pheatmap package in R software was performed to plot the heatmap of DEG (23).

### Gene ontology enrichment analysis

Gene ontology (GO) annotation contained three parts: BP, CC, and MF. For the GO and KEGG analyses, all candidate genes were mapped to each item in the Gene Ontology database^1^, and the number of genes in each item was calculated (24). Furthermore, hypergeometric test was applied to find the items that were significantly enriched in candidate genes compared with all background genes in this species. The *P* value is calculated using the base function phyper (version 4.3.0) of R software. Subsequently, multiple tests are carried out on *P* value to be positive, and the calibration software package is Q value (version 2.28.0).

### Gene set enrichment analysis

The gene set enrichment analysis (GSEA) software (version 4.0.3) was used on all detected genes with FDR < 0.05 as the criterion, and the top three of biological process (BP) and pathway enriched gene sets were selected for analysis. The gene sets in this analysis were collected from the Molecular Signatures Database (MSigDB, version 6.2)^2^ (25).

### Protein-protein interactions network construction and hub genes identification

Through the STRING database (version 11.5)^3^, we selected protein-protein interaction (PPI) analysis with a comprehensive score greater than 0.9 to construct a PPI network. Cytoscape (version 3.9.0) was used to visualize the PPI network and then analyzed the modules with higher scores in the PPI network through Molecular Complex Detection (MCODE). In addition, we used 12 algorithms in Cytohubba including MCC, DMNC, MNC, Degree, EPC, BottleNeck, Eccentricity, Closeness, Radality, Betweenness, Stress, and Clustering coefficient to calculate the total weight of each gene, and ultimately selected top 10 most frequently occurring 10 genes among the 12 algorithms as hub genes.

### Analysis of the connectivity map database

The connectivity map (CMAP) database^4^ is an open resource that links diseases, genes, and drugs through similar or opposite gene expression profiles (26). CMAP database was also used to predict the potential small molecule compounds that can reverse the changes in DEG expression. Mean ≤ 0.4 and *P* < 0.01 were set as the screening criteria.

## Results

### Baseline characteristics in salt-sensitive hypertension and salt-resistant hypertension patients

Whole transcriptome RNA-seq analysis was carried out on PBMCs isolated from five SSH and five SRH patients randomly selected from 72 patients.

The baseline characteristics of all patients with SRH and SSH are shown in Table 1. These include gender, age, blood pressure, heart rate, and so on. The results from two independent sample *t*-test showed that age, BMI did not significantly differ between the SSH and SRH groups by SPSS (version: SV26.0) (*P* > 0.05). In addition, we found significant differences in LDL-C, MCH, α-HBD (*P* > 0.05).

**TABLE 1:** Baseline characteristics of study participants in salt-sensitive hypertension (SSH) and salt-resistant hypertension (SRH).

Baseline content	SSH (*n* = 29)	SRH (*n* = 43)
Age (years)	56 ± 9	49 ± 10
Gender (Male, %)	34%	60%
Gender (Female, %)	66%	40%
SBP (mmHg)	150.31 ± 22.21	151.74 ± 19.35
SBP (mmHg)	89.55 ± 8.86	95.05 ± 14.96*
Heart rate	82.1 ± 13.46	83.18 ± 12.9
TBiL (μmol/L)	14.24 ± 4.68	14.13 ± 5.55
DBiL (μmol/L)	3.75 ± 1.1	3.7 ± 1.21
IBil (μmol/L)	10.49 ± 3.82	10.43 ± 4.87
AST (μ/L)	27.46 ± 9.75	25.65 ± 8.3
ALT (μ/L)	29.07 ± 23.96	25.74 ± 16.52
AST/ALT	1.23 ± 0.58	1.28 ± 0.7
ALP (μ/L)	72.96 ± 18.33	68.21 ± 16.09
MCH (pg)	28.46 ± 4.16	29.24 ± 2.46*
MCHC (g/L)	330.09 ± 11.99	334.66 ± 12.38

BMI, body mass index; SBP, systolic blood pressure; DBP, diastolic blood pressure; LDL, low-density lipoprotein, TBil, total bilirubin; DBiL, direct bilirubin; IBil, indirect bilirubin; AST, aspartate aminotransferase; ALT, alanine aminotransferase; ALP, alkaline phosphatase; MCH, mean corpuscular hemoglobin; MCHC, mean corpuscular hemoglobin concentration.

*P < 0.05 t-test.

Table 2 shows the saline-loading test of the study participants. The results show that SSH accounts for 36% and SRH for 64%.

**TABLE 2:** NS-loading test in salt-sensitive hypertension (SSH) and salt-resistant hypertension (SRH).

	SSH (*n* = 29)	SRH (*n* = 43)
SBP1	133.59 ± 16.83	147.07 ± 17.39
DBP1	82.21 ± 12.18	93.42 ± 12.26
SBP2	151.07 ± 19.58	140.02 ± 14.77
DBP2	91.97 ± 11.3	85.63 ± 12.46
PP1	51.28 ± 14.25	52.86 ± 16.27
PP2	59.1 ± 15.47	54.4 ± 10.1
MAP1	99.28 ± 12.24	111.49 ± 12.67
MAP2	111.76 ± 12.64	103.72 ± 12.43
MAP2- MAP1	12.37 ± 5.79	−7.71 ± 9.5

Before NS-loading test of MAP = MAP1.

After NS-loading test of MAP = MAP2.

Before NS-loading test of SBP = SBP1.

After NS-loading test of SBP = SBP2.

Before NS-loading test of DBP = DBP1.

After NS-loading test of DBP = DBP2.

Before NS-loading test of PP = PP1.

After NS-loading test of PP = PP1.

### Identification of differentially expressed genes

**FIGURE 1:**
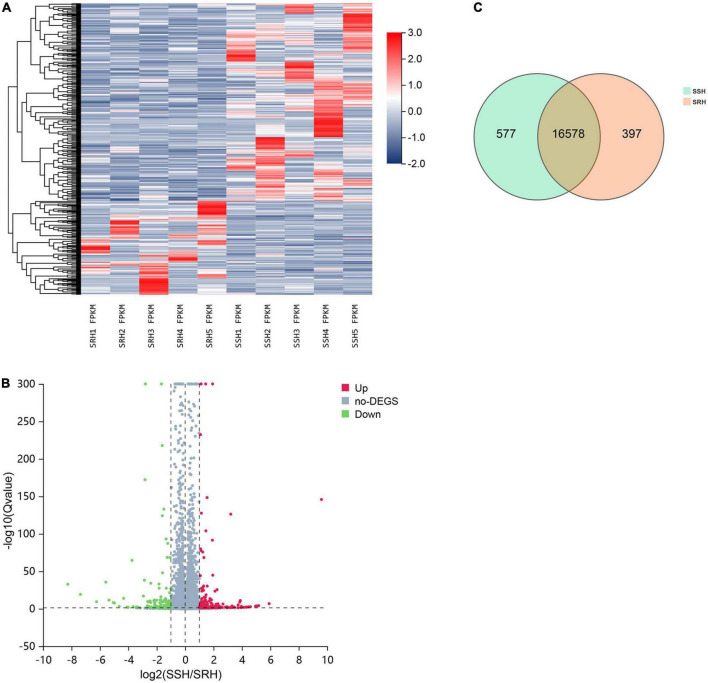
The discrepancy between salt-sensitive hypertension (SSH) and salt-resistant hypertension (SRH) in terms of gene expression profile. **(A)** Hierarchical cluster analysis of genes identified in SSH and SRH. **(B)** Distribution of the genes expressed in the two groups, red dots and green dots indicates genes significantly up-regulated or down-regulated in the SSH and SRH. **(C)** Number of Gene expressed in the two groups.

A total of 19,813 genes were identified in PBMC samples and 431 genes were found to be differentially expressed between SSH and SRH samples of which 294 were up-regulated and 137 down-regulated genes in the SSH group. The criteria for DEGs were selected to q-value < 0.05 and |log2Foldchange| > 1. The heatmap and volcano plots for DEGs are shown in Figures 1A,B. The Venn diagram shows the number of genes unique and shared between the two groups. The shared number of genes shared between SSH and SRHare16578 (Figure 1C).

### Gene ontology and Kyoto encyclopedia of genes and genomes pathway analysis of differentially expressed genes

**FIGURE 2:**
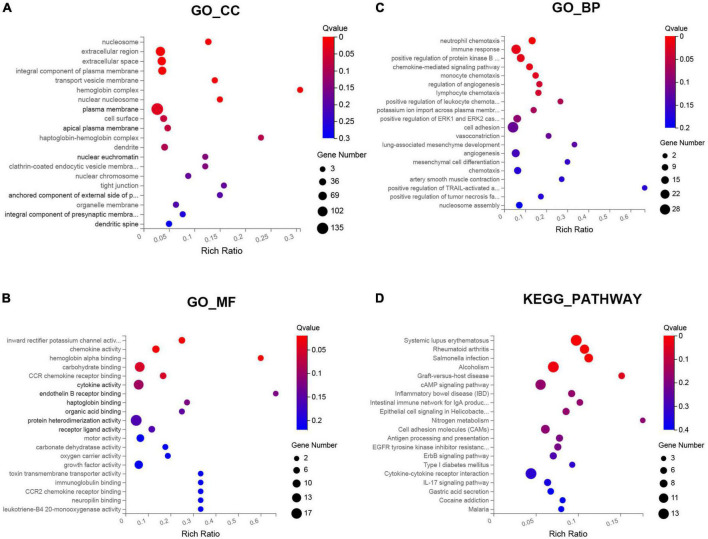
Gene ontology (GO) and Kyoto encyclopedia of genes and genomes (KEGG) pathway enrichment analysis of the differentially expressed genes (DEGs) between SSH and SRH in the biological processes (BPs), cellular components (CCs), and molecular functions (MFs). **(A)** GO-CC, **(B)** GO-MF, **(C)** GO-BP, and **(D)** KEGG pathway.

Gene ontology functional enrichment analysis for these DEGs were performed. The results indicated that these DEGs were significantly enriched in eight enriched cellular component (CC), such as plasma membrane, nuclear nucleosome, and hemoglobin complex (Figure 2A). For molecular functions (MF), these DEGs were mainly enriched in hemoglobin alpha binding, chemokine activity, and inward rectifier potassium channel activity (Figure 2B). In addition, these DEGs were most significantly enriched in the BP of immune response, cell adhesion, and inflammatory chemotaxis (Figure 2C). The enriched KEGG pathways were performed in the top five signal pathways, including Rheumatoid arthritis, Systemic lupus erythematosus, Salmonella infection, Alcoholism, and Graft-versus-host disease (Figure 2D).

### Gene set enrichment analysis

**FIGURE 3:**
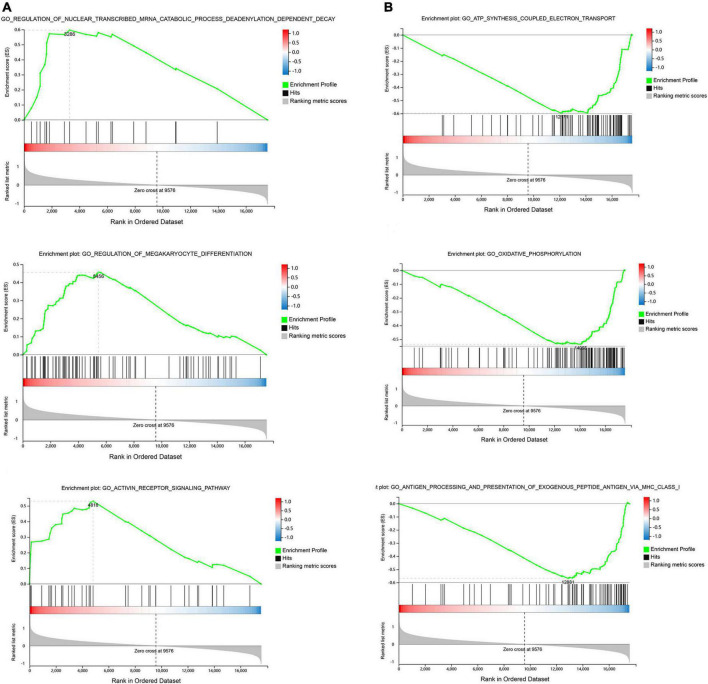
Enrichment analyses via gene set enrichment analysis (GSEA). **(A)** The top three enriched up-regulated GO gene sets. **(B)** The top three enriched down-regulated GO gene sets.

**FIGURE 4:**
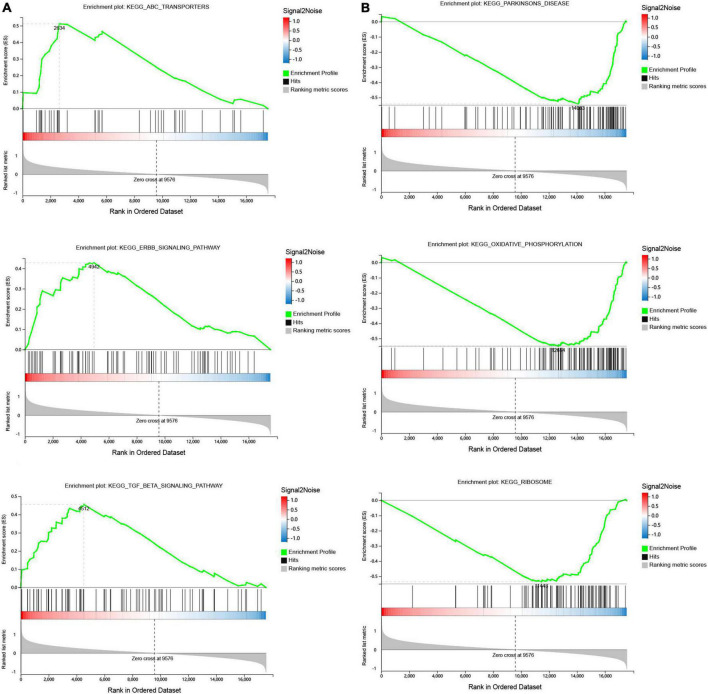
Gene set enrichment analysis (GSEA) of differentially expressed genes (DEGs) using the GSEA Kyoto encyclopedia of genes and genomes (KEGG) pathways database. **(A)** The top three enriched up-regulated KEGG pathway sets. **(B)** The top three enriched down-regulated KEGG pathway sets.

Gene set enrichment was performed to identify gene sets with statistically significant differences between the SSH group and the SRH group. The top three of GO_BP sets are shown. The most significant enriched up-regulated GO_BP sets included regulation of nuclear-transcribed mRNA catabolic process deadenylation dependent decay, Regulation of megakaryocyte differentiation, and Receptor signaling pathway (Figure 3A). The most significantly enriched down-regulated GO_BP sets included ATP synthesis coupled electron transport, Oxidative phosphorylation, Antigen processing, and presentation of exogenous peptide antigen via MHC class I (Figure 3B). As illustrated in Figure 4, to determine signaling pathways associated with SSH and SRH, GSEA pathway analysis was performed on DEGs. The top three most significantly enriched gene sets positively correlated with the SSH group were ABC transporters, ERBB signaling pathway, and TGF beta signaling pathway (Figure 4A). The top three most significantly enriched gene sets negatively correlated with the SSH group were PARKINSONS disease, oxidative phosphorylation, and ribosome (Figure 4B).

### Protein-protein interaction network construction and hub gene identification

**FIGURE 5:**
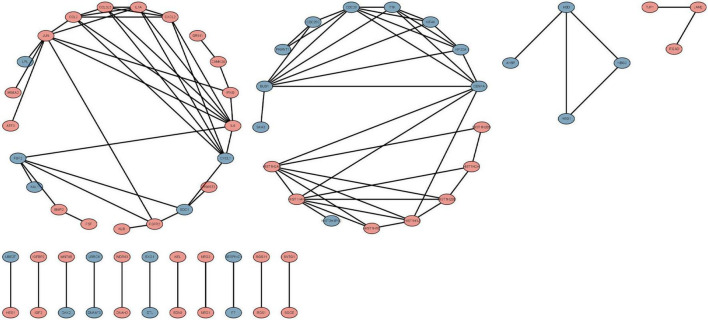
The protein-protein interaction (PPI) network of differentially expressed genes (DEGs). Circles and lines represent genes and the interaction of proteins between genes, respectively. The red represents the up-regulated genes. The blue represents the down-regulated genes.

**FIGURE 6:**
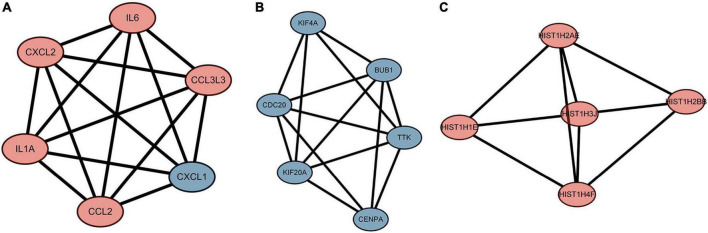
The highly expressed protein-protein interaction (PPI) network. **(A)** Score 6, **(B)** Score 5.6, **(C)** Score 4.6. The red represents the up-regulated genes. The blue represents the down-regulated genes.

**FIGURE 7:**
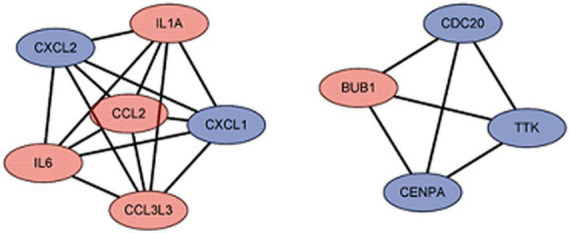
The hub gene of differentially expressed genes (DEGs). The red represents the up-regulated genes. The blue represents the down-regulated genes.

**FIGURE 8:**
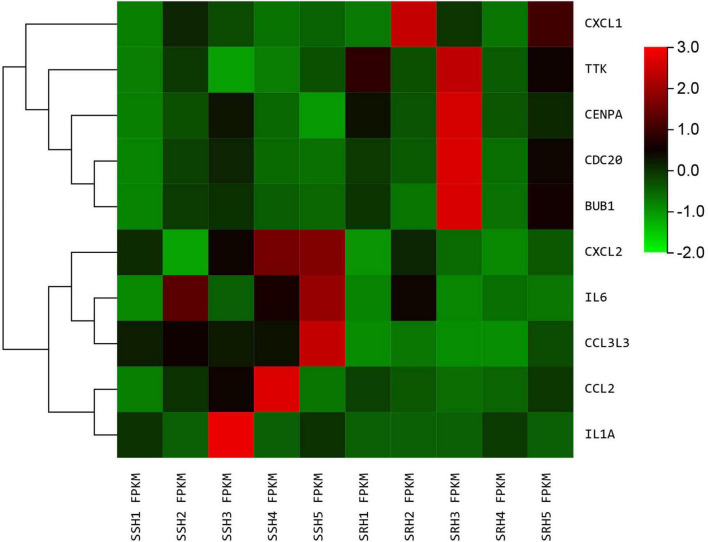
The heatmap of hub genes. Color from green to red indicated low to high representation value.

We used the STRING database to construct the PPI network, which consisted of 396 nodes and 89 edges (Figure 5). Nodes represent DEGs, and edges represent interactions between DEGs. High-scoring modules in the PPI network were analyzed by MCODE (Figure 6). The ten identified hub genes (IL6, CCL2, IL1A, CXCL1, BUB1, CCL3L3, CDC20, CENPA, CXCL2, and TTK) were showed in Table 3 and Figure 7. The expression level of the hub gene in samples were shown in Figure 8.

**TABLE 3:** Top ten genes in the 12 statistical methods.

Number	Gene	The number of occurrences	Expression
1	IL6	10	Up
2	CCL2	10	Up
3	IL1A	8	Up
4	CXCL1	8	Down
5	BUB1	8	Up
6	CCL3L3	7	Up
7	CDC20	7	Down

### Analysis of the connectivity map database

Analysis of the CMAP database showed that iopromide and iloprost compounds potentially exerted a negative regulatory effect on DEGs (Table 4). We deduced that these two drugs are potential candidates to alleviate SRH progression to SSH.

**TABLE 4:** The result of connectivity map (CMAP) database analysis.

Rank	CMAP name	Mean	*n*	Enrichment	*p*	Specificity	Percent non-null
1	Metitepine	0.577	4	0.827	0.00135	0.0051	75
2	Iopromide	−0.301	4	−0.804	0.00284	0.0074	50
3	Pipemidic acid	0.364	3	0.873	0.00373	0.0159	66
4	Iloprost	−0.375	3	−0.876	0.00385	0.0063	66
5	Cephaeline	0.509	5	0.716	0.00427	0.3151	80
6	Securinine	0.455	4	0.762	0.00605	0.0325	75
7	Lycorine	0.455	5	0.695	0.00655	0.1235	80
8	Thiostrepton	0.343	4	0.737	0.00939	0.098	50

## Discussion

Two long-term follow-up investigations revealed that SSH patients had a greater risk of cardiovascular events and death than SRH patients (27, 28). Early identification and management in high-risk individuals are challenging due to a lack of good biomarkers and mechanistic investigations on SSH. PBMCs in peripheral blood are easy to acquire and may exhibit changes in blood pressure, making them excellent for gene expression research. To date, the current study is the most comprehensive study where the first time we used RNA-seq technology in PBMC to describe the global transcriptome of differential genes between SSH and SRH patients. Therefore, we employed RNA-seq to investigate SSH biomarkers and potential intervention targets, which could provide new insights into the molecular mechanisms of SSH.

In the current study, we used RNA-seq to evaluate 294 up-regulated and 137 down-regulated genes in PBMCs, each of which had the fold change > 1.0 and with a value of q < 0.05. Moreover, all the detected DEGs were significantly enriched in BP such as immune response, inflammatory chemotaxis, and cell adhesion. A recent study has revealed that inflammation is a part of the etiology of hypertension that can make changes in the immune system which ultimately disrupt the regulatory functions of these systems and eventually unbalancing the homeostatic regulation of blood pressure (29). The results of our study indicated that the DEGs were significantly enriched in CC such as plasma membrane, nuclear nucleosome, and hemoglobin complex. In essential hypertension, sodium and calcium transport across plasma membranes is critical. Earlier it has been reported that plasma membrane defect precedes the development of primary hypertension (30). For the MF, DEGs were mainly enriched in hemoglobin alpha binding, chemokine activity, and inward rectifier potassium channel activity. The chemotaxis of immune-inflammatory cells to the blood vessel wall is a significant process of the immune inflammatory response in hypertension as chemokines and their receptors are the key factors mediating the chemotaxis of immune-inflammatory cells. Chemokine Receptor CXCR2-positive cells were discovered to be substantially more in hypertension patients’ blood than in healthy persons (31). The KEGG results showed that DEGs were significantly enriched in cytokine-cytokine receptor interaction, IL-17 signaling pathway, and CAMP signaling pathway. The reciprocal control of cytokines via synthesis and secretion has a function in the onset and progression of hypertension. It has been demonstrated the secretion of cytokines is associated with catecholamines such as epinephrine, and limiting their synthesis can effectively control the occurrence of cytokine release syndrome (32). This could be milestone to treat hypertension. In addition, GSEA identified TGF signaling pathway as the significantly enriched pathway in SSH Where the TGF signaling pathway plays a crucial role in both angiogenesis and pathological conditions, and it has a long-recognized role in hypertension (33, 34).

Next, we analyzed the PPI network and hub genes of the two groups including pro-inflammatory cytokines such as IL-1A, IL-6, chemokines, and receptors such as CCL2, CXCL2, CXCL1, and CCL3L3 which suggested that interactions between cytokines may have a regulatory role in SSH. There is increasing evidence that hypertension is an inflammatory condition. Notably, mediators released by immune cell activation promotes hypertensive target organ damage (35). Both IL-1 isoforms IL-1α and IL-1β correlated with the degree of blood pressure regulation. Earlier it was also reported that IL-1 receptor deficiency or blockade also linked to reduce the hypertension (36, 37). Activation of monocytes in PBMCs is associated with peripheral hypertension and higher levels of plasma pro-inflammatory cytokines (38). Previous research has found that the majority of chemokines belong to the CC and CXC families. It has been observed that the rise of cytokines such as IL-6 and CCL2 in the high-salt diet-induced hypertension rat model is connected with the NLRP3 inflammatory mechanism (39). Moreover, chemokines can partially regulate the movement of PBMCs, and chemokines of the CXC family may have mutual regulation in hypertension and target organ damage, which may provide new insights into chemotactic immunotherapy for hypertension (40). Therefore, the role of another hub gene construction has not been reported to be related to hypertension that might be new targets for SSH.

In modern drug research, the discovery of new gene targets is often the premise of new drug innovation. To link genes to drugs, we further scanned for some potential small-molecule compounds and using the CMAP database we identified two small molecular compounds (iopromide and iloprost) that can potentially interact with DEGs. CMAP database for prediction of potential drugs may reverse expression of SSH-related genes. According to studies, iloprost has adequate pulmonary vasodilation and can improve symptoms and exercise capacity in people with primary pulmonary hypertension (41, 42). These small molecules might be investigated as potential potential therapeutic targets for the treatment of SSH. Of course, this research limited in the PBMCs and is not necessarily applicable to all cells. In the future, we will also conduct experiments to verify these targets on a bigger sample-sized population.

## Conclusion

In the present study, we identified 431 DEGs and 10 hub genes by RNA-seq from PBMCs of SSH and SRH. In conclusion, our study found several potential biomarkers and provided a comprehensive understanding of SSH. Additionally, we identified that the iopromide and iloprost could be the potential therapeutic targets for the treatment of SSH.

## Data availability statement

The datasets presented in this study can be found in online repositories. The names of the repository/repositories and accession number(s) can be found in the article/supplementary material.

## Ethics statement

The studies involving human participants were reviewed and approved by the Medical Ethics Committee of The First Affiliated Hospital of Guangxi Medical University. The patients/participants provided their written informed consent to participate in this study.

## Author contributions

XC wrote the first draft of the manuscript. ZJ wrote a section of the manuscript. RH and GZ guided the writing. All authors listed have made a substantial, direct, intellectual contribution to the work, reviewed the final manuscript, and approved it for publication.

## Footnotes

1
http://www.geneontology.org/

2
http://www.broadinstitute.org/gsea/msigdb/

3
http://string-db.org/

4
https://clue.io/
